# Isolation, molecular identification, and pathological lesions of *Saprolegnia* spp. isolated from common carp, *Cyprinus carpio* in floating cages in Mosul, Iraq

**DOI:** 10.14202/vetworld.2020.2759-2764

**Published:** 2020-12-24

**Authors:** Fawwaz Fadhil Ali, Shahbaa Khalil AL-Taee, Zahraa Mustafa AL-Jumaa

**Affiliations:** 1 Department of Animal Production, Institute of Mosul, Northern Technical University, Mosul, Iraq; 2 Department of Pathology and poultry diseases, College of Veterinary Medicine, Mosul, Iraq; 3 Department of Internal and Preventive Medicine, College of Veterinary Medicine, Mosul, Iraq

**Keywords:** common carp, fungal isolation, histopathological changes, molecular identification, *Saprolegnia*

## Abstract

**Background and Aim:**

Saprolegniasis is a fungal disease that infects freshwater fish. The condition is characterized by a cotton-like appearance in the gills and body. This study aimed to isolate *Saprolegnia* from common carp, *Cyprinus carpio*, raised in a floating cage in Wana district, Mosul, Iraq.

**Materials and Methods:**

Samples were collected from 15 infected fish and examined microbiologically, molecularly, and histopathologically. *Saprolegnia* DNA was extracted which was amplified using universal primers give a 540 bp DNA fragment, and gill and muscle tissue were also examined for histopathological changes.

**Results:**

Isolated colonies of *Saprolegnia* were characterized by a circular, white cottony appearance with long hair. Lactophenol staining demonstrated hyphae as branched non-septate, transparent masses. The genomic DNA of isolates was consistent with *Saprolegnia* spp. The infected tissue samples showed variable pathology in gills. Severe hemorrhage and edema were observed in primary gill filaments with hyperplasia in epithelial cells and infusion in secondary gill filaments. Hyphae of *Saprolegnia* were seen between necrotic and edematous myofiber with inflammatory cells infiltration.

**Conclusion:**

*Saprolegnia* can cause economic impacts through lethal infection of fish. Clinical signs of *Saprolegnia* infection were confirmed molecularly and microscopically, and these findings were supported by histopathological lesions in gill and muscle tissues.

## Introduction

The eukaryotic oomycetes fungi are pathological microorganisms responsible for infections in both animals and plant infections [1]. Severe infections in fish are known as saprolegniosis disease [2,3]. Oomycetes fungi cause significant economic loses to fish production, including salmon, trout, and catfish, which are more susceptible to oomycete infection [4]. Infection is also observed in zebrafish and silver crucian carp [5,6]. Fish pathogens are mostly classified in the order, Saprolegniales, and eight genera infect fish, either naturally or artificially, including *Calyptratheca*, *Leptolegnia*, *Achlya*, *Aphanomyces*, *Leptomitus*, *Pythiopsis*, *Thraustotheca*, and *Saprolegnia*. *Saprolegnia*, *Aphanomyces*, and *Achlya* are important pathogens for aquaculture [7,8]. Infected fish display different levels of susceptibility but manifest the same symptoms [9]. *Saprolegnia parasitica* is responsible for substantial fish mortality [10]; *Saprolegnia* spp. infections are closely associated with catastrophic losses in fish production in both freshwater and marine aquaculture industries [11]. Pathogenic oomycetes also infect fish eggs and cause considerable losses in fish hatcheries [12].

The oomycete infection occurs by translocation of effector proteins into infected cells; these proteins impede host defenses [13]. *S. parasitica* is a fish pathogen with secondary cysts characterized by clusters of long-haired hook bundles. These hooks, along with adhesive extracellular matrix and proteins (fibronectin and thrombospondin), increase the strength of cyst attachment to hosts [14], and leading to damage the host epidermis cells and consequently causes tissue damage and dehydration as a result of the release of toxic materials and body fluids [9].

No effective treatments are available for *Saprolegnia* infection [15]. However, some medications, such as antimicrobial peptides that enhance host immune response, have been tried [4]. Further, clotrimazole could be effective against *Saprolegnia* infections through pretreatment of fish eggs of freshwater-farmed fish [16]. In addition to Virkon® S, it has been shown to control and prevent saprolegniasis infection in common carp [17].

*Saprolegnia* causes considerable impact in local fish industries, particularly in Mosul city in North Iraq. However, limited studies are available to characterize the pathogen in this region. This study was aimed, primarily, at identification of the pathogen using microbiological, molecular, and histopathological methods. This investigation will help identify and confirm this disease’s environmental distribution, thus informing development of measures to control the disease.

## Materials and Methods

### Ethical approval

In this study, ethical approval was not required; however, samples were collected based on the standard procedure and institutional guidelines for sample collection.

### Study period and location

The infected fish were collected from different sites from floating cages in the Wana sub-district in Mosul, Iraq, from March to July 2019.

### Fish

**Figure-1:**
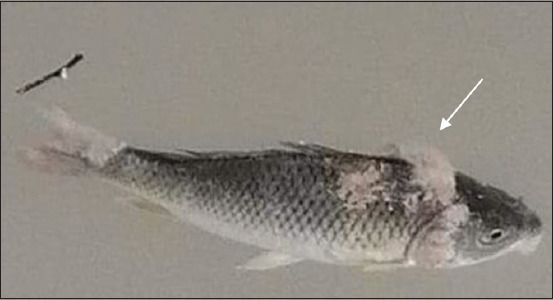
*Cyprinus carpio* infected with *Saprolegnia*, the diseased fish exhibit the characteristic manifestation of water fungus infection. The arrow is pointed to cottony wool-like lesions on fish skin.

A 15 infected common carp “*Cyprinus carpio*” from floating cages in Wana/Mosul, Iraq, were investigated. Infected fish were examined, and samples were collected from fish that exhibited Saprolegniosis signs [18], including cotton wool-like lesions on the head, fin, and body, skin ulceration, and loss of appetite, Figure-1. These fish were placed in polyethylene bags and transported under sterile conditions to laboratories at the College of Veterinary Medicine, Mosul University, Mosul, Iraq.

### Microbiological examination of the collected samples

All samples were collected and immediately transported in cold conditions. These samples were cultured on Sabouraud’s dextrose agar (SDA) plates. Medium was composed of 65 g of SDA base with 250 mg chloramphenicol, 26 mg of gentamycin, 5 g of yeast extract, and the distilled water constituents and adjust volume 1 L. After dissolution, pH was adjusted to 5.8. The inoculated plates were incubated at 20-25°C for 5-10 days. Plates without positive growth were further incubated for up to 4 weeks before discarding. All positive cultures were subcultured using SDA plates to obtain pure axenic single colonies. These plates were incubated at 20-25°C for 3-5 days. For microscopic examination, slides were prepared from each colony using a tape method. Transparent tape was lightly pressed onto growing colonies. The tape was fixed over a clean slide with a drop of lactophenol cotton blue stain. Stained slides were observed under a microscope at 50× and 200× to identify this fungus, using a fungal identification key [19]. Shape and diameter of hyphae and spores from isolated fungi were measured. All purified cultures were examined for macro- and micromorphological characteristics. Gross morphological examination recorded the rate of the fungal growth, texture, changes in color during growth, the final color of surface, and reverse sides of the colonies.

### DNA analysis

The DNA of *Saprolegnia* was extracted directly from infected fish tissues according to the manufacturer instructions (gSYNC™ Geneaid Extraction Kit). Briefly, collected samples were prepared and DNA extracted following kit manual instructions. All extracted DNAs were stored at −20°C until analyzed. Molecular identification of *Saprolegnia* used the internal transcribed spacer (ITS) region. Sequencing used universal primers ITS1 (TCCGTAGGTGAACCTGCGG) and ITS4 (TCCTCCGCTTATTGATATGC). Two pairs of primers (forward and reverse) were synthesized by BIONEER Co. (Korea) for targeting the fungus. Polymerase chain reaction (PCR) reaction used a 25 μL reaction volume and described in Table-1. The amplification program is specified in Table-2.

**Table-1:** Final polymerase chain reaction volume composition (total volume of 25 μL).

Content	Amount (μL)
Forward primer (10 picomol/μL)	1
Reverse primer (10 picomol/μL)	1
Template DNA 250 ng/μL	5
DDW	6.5
MgCl_2_	1.5
Mstermix 2.5 ×	10
Total volume	25

Figures in parentheses are indicated as percentages

**Table-2:** Polymerase chain reaction setting program of amplification.

Number of cycles	Adjusted temperature (°C)	Time	Discretion
1	95	5 min	Initial DNA denaturation
30	95	20 s	DNA denaturation
	57	30 s	Primer annealing
	72	30 s	Primer extension
1	72	5 min	Final extension
1	4	Hold	Cooling

All PCR products were analyzed using 2% agarose gel electrophoresis (Biometra, Germany), with 0.2 μL ethidium bromide in TBE buffer. DNA bands were visualized with a UV transilluminator.

### Histological examination

Different infected tissues included gill affected by sloughing and necrotic appearance and muscle with ulceration. The selected tissues were separated and fixed in 10% neutral buffered formalin for 48-72 h. Samples were then embedded in paraffin, and 5 μm tissue sections were cut using a microtome. The tissue sections were then fixed on slides and stained with hematoxylin and eosin stain [20-22].

## Results

### Saprolegniosis disease symptoms and colonies morphology

**Figure-2:**
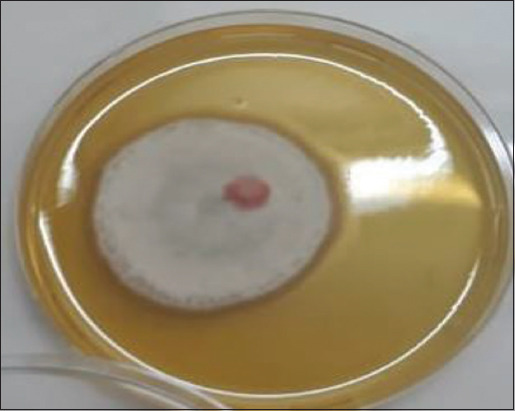
Macroscopic morphology of isolated *Saprolegnia* spp., the macroscopic appetence, and characteristics of isolated fugues, the positive *Saprolegnia* spp. colony (Sabouraud’s dextrose agar) after 14 days of incubation at 25°C. The colony appeared as a circular cottony with long hairs and white.

The infected common carp showed loss of appetite and a white to gray cotton-like growth cover on fish skin, fins, and gills (Figure-1). Cultured fungus on SDA plates showed characteristic morphology and proper growth of *Saprolegnia*. Colonies start to be observed within 4 days of incubation. These colonies are circular with a cottony appearance, white in color, with long hairs (Figure-2). This evidential characteristic of diseased common carp and colony morphology indicated *Saprolegnia* infection.

### Microscopic examination

**Figure-3:**
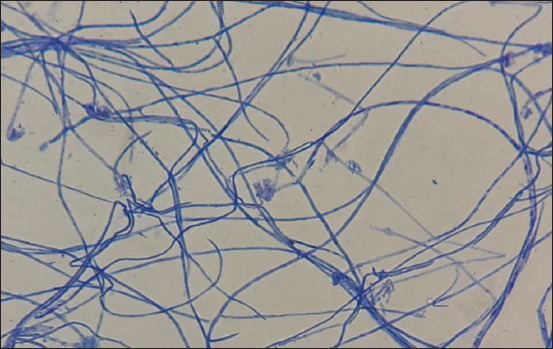
The microscopic characterization of isolated fungus. The microscopic inspection of the isolated fungus showed the branched non-septate hyphae of *Saprolegnia* isolate, together with masses (different in length and width), transparent and has a cell membrane stained with lactophenol cotton blue stain. These images were obtained using a 40× objective lens.

The microscopic examination revealed branched non-septate, transparent masses varying in length and width. Hyphae of *Saprolegnia* isolates stained with lactophenol cotton blue stain (Figure-3).

### PCR analysis

**Figure-4:**
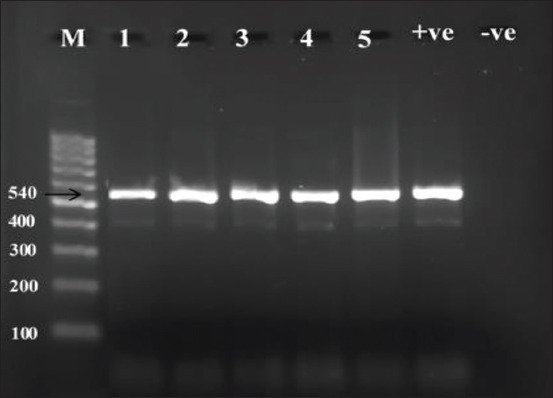
The polymerase chain reaction (PCR) amplification products of TSI on 2% agarose gel. The TSI was amplified from extracted DNA using the condition described in materials and methods. Four microliters of PCR amplification product were mixed with 1 μL of loading buffer for DNA electrophoresis, and then, the mixture was loaded for each well of 2% agarose gel. The DNA size was indicated using the DNA ladder. M: Marker (100-1000 bp). 1-5: Sample numbers that showed positive at 540 bp. +ve: Positive control, positive and -ve: Negative control.

The extracted DNA was amplified to identify *Saprolegnia* spp. molecularly using universal primers. The amplification PCR products showed the target identified 540 bp DNA fragment (Figure-4), which indicates the fungal *Saprolegnia*. All 50 samples were analyzed and present positive results.

### Histological examination

#### Gills

**Figure-5:**
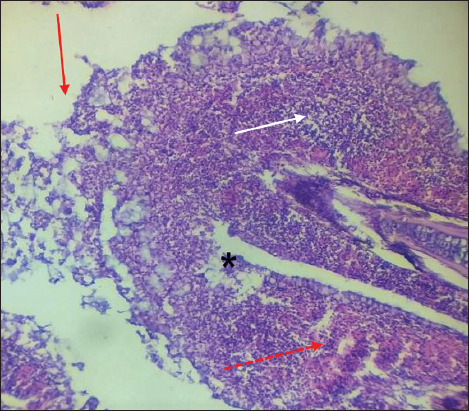
Gills of *Cyprinus carpio* infected with *Saprolegnia* exhibit sloughing of a pix of primary gill filament (red row), severe hemorrhage (red dot row) with edema (black star), and infiltration of inflammatory cells (white row). H&E.

**Figure-6:**
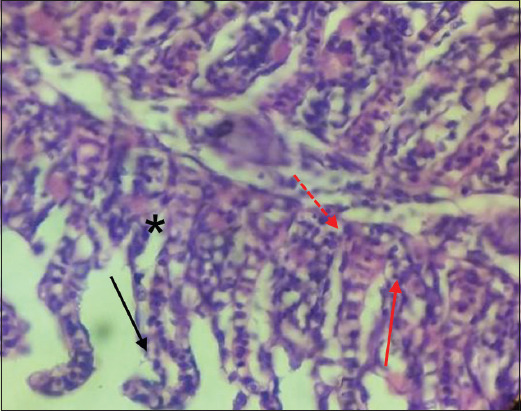
Gills of *Cyprinus carpio* infected with *Saprolegnia* exhibit partial occlusion of interlamellar spaces (red row), hyperplasia of epithelial gill filament (red dot row) with edema (black star), and infusion of secondary gill filament (black row). H&E, 1×40.

**Figure-7:**
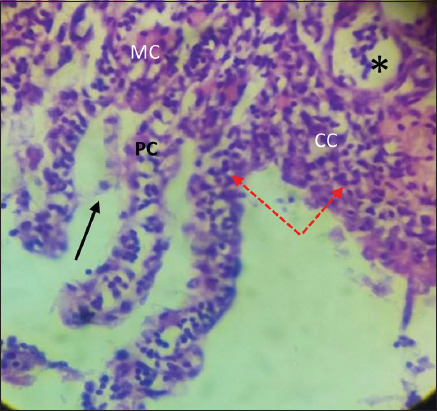
Gills of *Cyprinus carpio* infected with *Saprolegnia* exhibit hypertrophy of mucus cells, chloride cells, and pillar cells with edema (black star) and lifting epithelial cells (black row), hemorrhage (red row) with infiltration of inflammatory cells (red dot row) H&E, 1×40.

The infected gills showed sloughing of primary gill filaments, severe hemorrhage with edema, and infiltration of inflammatory cells (Figure-5). This infected tissue showed other histopathological changes, such as hyperplasia of epithelial cells, infusion of secondary gill filaments and partial occlusion interlamellar space (Figure-6). Besides, the hypertrophy of mucus, chloride, and pillar cells combined with hemorrhage, edema, and lifting epithelial cells (Figure-7).

#### Muscles

**Figure-8:**
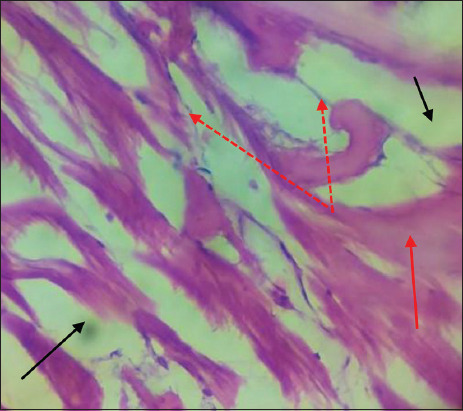
Muscles of *Cyprinus carpio* infected with *Saprolegnia* exhibit necrosis of muscle fiber (red row), edema (black row) with penetrating fungal hyphae were observed in muscle fiber (red dot row) H&E, 1×40.

**Figure-9:**
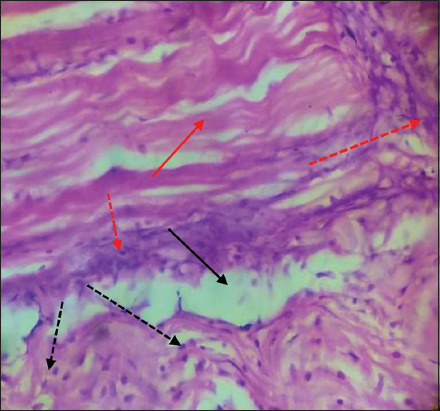
Muscles of *Cyprinus carpio* infected with *Saprolegnia* exhibit necrosis of muscle fiber (red row), edema (black row) with penetrating severe fungal hyphae were observed in muscle fiber (red dot row) moderate infiltration of inflammatory cells (black dot row) H&E, 1×40.

Microscopic examination of infected muscle exhibited myofibril degeneration and necrosis with edema. Penetrating fungal hyphae into the muscle layer was also observed (Figure-8) in addition to moderate infiltration of inflammatory cells with severe fungal hyphae penetration into muscle fibers (Figure-9).

## Discussion

The fish industry in Iraq has grown considerably [23]. About 1,074,000 hectares of water resources, including revisers, lakes, reservoirs, and other resources, are involved in fish production [24]. The spread of fish disease is increasing challenges and economic losses, particularly for increasing fish mortality [25,26].

This research identifies the oomycete fungus, *Saprolegnia*, in isolates from common carp *C. carpio* from floating cages in Wana/Mosul, Iraq. Classical and molecular-based analyses were applied, supported by histopathology. Samples were collected from fish infected with *Saprolegnia* spp. which were identified based on symptoms, physiological characteristics, molecular analysis, and histopathological changes.

The oomycetes fungus, *Saprolegnia*, is pervasive in freshwater, and it is the significant cause of freshwater fungal infection of fish and eggs [27] and is the main cause of saprolegniasis [28]. This disease causes high fish mortality, particularly in winter [29]. Infected *C. carpio*show characteristic cotton-like growth on skin, gills, and fins (Figure-1). *Saprolegnia* infects and penetrates the fish epidermis. The infection begins on fins or heads and spreads to other parts of fish bodies [30,31]. The fungus was isolated using SDA medium. Different media have been used to separate this fungus, including MEA, PDA, and SDA, with varying growth rates and areal mycelium formation [32]. Culture characteristics supported by PCR effectively confirmed *Saprolegnia* infection [33]; non-coding ITS regions ITS1 of rDNA are widely accepted genetic markers because of their relatively high sequence variability and the availability of primers for amplification of fungal sequences. These ITS regions are located between two coding regions, the 18S and 28S genes. Molecular analysis of these regions was adopted to confirm *Saprolegnia* isolates [34,35].

Histopathological changes in gills of infected fishes showed sloughing of primary gill filaments, combined with edema and severe hemorrhage with signs of inflammation. These histopathological changes are often reported [36-38]. Examination of infected muscle tissue showed degeneration and necrosis of myofibrils. Such pathology is also reported previous investigation of *Saprolegnia* infection [17,39,40]. Lesions caused by oomycetes disease include loss of integument integrity, degeneration of infected muscles, and edema. Deep penetration into muscle fiber is observed in severe infections with focal cellular necrosis [39]. Intracellular epidermal edema and epidermal sloughing enhance basement membrane penetration by the fungal hyphae [41]. Penetration of fungal hyphae to the muscle layer was also observed. Such damage is also reported previously in *Saprolegnia* infection [17,39,41,42].

## Conclusion

We conclude that *Saprolegnia* spp. was isolated and confirmed molecularly in isolates from infected common carp in floating cages in Wana district, Mosul, Iraq. The histopathological lesions in infected fish gills and muscles support this evidence. Further investigation is needed to define the environmental distribution of infected fungal-like pathogens and other related mycotic pathogens in fish farms in Iraq, thus facilitating the design of mycotic infection control program to reduce or eliminate economic losses.

## Authors’ Contributions

SKA and ZMA accomplished sample collection and experimental design; FFA analyzed, interpreted the data and drafted the manuscript, all authors involved in manuscript reading and editing. All authors read and approved the final manuscript.

## References

[1] Wuensch A, Trusch F, Iberahim N.A, van West (2018). *Galleria mellonella* as an experimental *in vivo* host model for the fish-pathogenic oomycete *Saprolegnia parasitica*. Fungal Biol.

[2] van West P, de Bruijn I, Minor K.L, Phillips A.J, Robertson E.J, Wawra S, Bain J, Anderson V.L, Secombes C.J (2010). The putative RxLR effector protein SpHtp1 from the fish pathogenic oomycete *Saprolegnia parasitica* is translocated into fish cells. FEMS Microbiol. Lett.

[3] Rzeszutek E, Diaz-Moreno S.M, Bulone V (2019). Identification and characterization of the chitin synthase genes from the fish pathogen *Saprolegnia parasitica*. Front. Microbiol.

[4] de Bruijn I, Belmonte R, Anderson V.L, Saraiva M, Wang T, van West P, Secombes C.J (2012). Immune gene expression in trout cell lines infected with the fish pathogenic oomycete *Saprolegnia parasitica*. Dev. Comp. Immunol.

[5] Ke X, Wang J, Gu Z, Li M, Gong X (2009). *Saprolegnia brachydanis*, a new oomycete isolated from zebrafish. Mycopathologia.

[6] Ke X.L, Wang J.G, Gu Z.M, Li M, Gong X.N (2009). Morphological and molecular phylogenetic analysis of two *Saprolegnia* sp. (*Oomycetes*) isolated from silver crucian carp and zebrafish. Mycol. Res.

[7] Hatai K, Hoshiai G.I (1993). Characteristics of two *Saprolegnia* species isolated from coho salmon with saprolegniosis. J. Aquat. Anim. Health.

[8] Bly J.E, Lawson L.A, Abdel-Aziz E.S, Clem L.W (1994). Channel catfish, *Ictalurus punctatus*, immunity to *Saprolegnia* sp. J. Appl. Aquac.

[9] Tiffney W.N (1939). The host range of *Saprolegnia parasitica*. Mycologia.

[10] Rocchi S, Tisserant M, Valot B, Laboissière A, Frossard V, Reboux G (2017). Quantification of *Saprolegnia parasitica* in river water using real-time quantitative PCR:From massive fish mortality to tap drinking water. Int. J. Environ. Health Res.

[11] Tedesco P, Beraldo P, Massimo M, Fioravanti M.L, Volpatti D, Dirks R, Galuppi R (2020). Comparative therapeutic effects of natural compounds against *Saprolegnia* spp. (Oomycota) and *Amyloodinium ocellatum* (*Dinophyceae*). Front. Vet. Sci.

[12] Earle G, Hintz W (2014). New approaches for controlling *Saprolegnia parasitica*, the causal agent of a devastating fish disease. Trop. Life Sci. Res.

[13] Wawra S, Bain J, Durward E, de Bruijn I, Minor K.L, Matena A, Löbach L, Whisson S.C, Bayer P, Porter A.J, Birch P.R.J, Secombes C.J, van West P (2012). Host-targeting protein 1 (SpHtp1) from the oomycete *Saprolegnia parasitica* translocates specifically into fish cells in a tyrosine-O-sulphate-dependent manner. Proc. Natl. Acad. Sci. U. S. A.

[14] Rezinciuc S, Sandoval-Sierra J.V, Ruiz-León Y, van West P, Diéguez-Uribeondo J (2018). Specialized attachment structure of the fish pathogenic oomycete *Saprolegnia parasitica*. PLoS One.

[15] Belmonte R, Sandoval-Sierra J.V, Ruiz-León Y.V, Diéguez-Uribeondo J (2014). Role of pathogen-derived cell wall carbohydrates and prostaglandin E2 in immune response and suppression of fish immunity by the oomycete *Saprolegnia parasitica*. Infect. Immun.

[16] Warrilow A.G, Hull C.M, Rolley N.J, Parker J.E, Nes W.D, Smith S.N, Kelly D.E, Kelly S.L (2014). Clotrimazole as a potent agent for treating the oomycete fish pathogen *Saprolegnia parasitica* through inhibition of sterol 14alpha-demethylase (CYP51). Appl. Environ. Microbiol.

[17] Ashour A.A, Mustafa S. A, Yassein S.N (2017). Histopathological Studies on Common Carp *Cyprinus carpio* L. Infected with *Saprolegnia* sp, Treated with Virkon^®^.

[18] Peyghan R, Rahnama R, Dezfuly Z.T, Shokoohmand M (2019). *Achlya* infection in an oscar (*Astronotus ocellatus*) with typical symptoms of saprolegniosis. Vet. Res. Forum.

[19] Ellis D, Davis S, Alexiou H, Handke R, Bartley R (2007). Descriptions of Medical Fungi. Nexus Print Solutions, Adelaide, South Australia, Australia.

[20] Roberts R.J (2001). Fish Pathology.

[21] Abdel-Latif H.M.R, Abdel-Tawwab M, Khafaga A.F, Dawood M.A.O (2020). Dietary *Origanum* essential oil improved antioxidative status, immune-related genes, and resistance of common carp (*Cyprinus carpio* L.) to *Aeromonas hydrophila* infection. Fish Shellfish Immunol.

[22] Abdel-Latif H.M.R, Abdel-Tawwab M, Khafaga A.F, Dawood M.A.O (2020). Dietary oregano essential oil improved the growth performance via enhancing the intestinal morphometry and hepato-renal functions of common carp (*Cyprinus carpio* L.) fingerlings. Aquaculture.

[23] Al-Salem A.F.B (2013). Technical and economical evaluation of fish cage culture projects in Babylon Province. Al-Musaib Technical College.

[24] Mhaisen F.T (1993). A review on the parasites and disease in fish ponds and farms of Iraq. Iraqi J. Vet. Sci.

[25] Gjedrem T (2015). Disease resistant fish and shellfish are within reach:A review. J. Mar. Sci. Eng.

[26] Abdel-Latif H.M.R, Dawood M.A.O, Menanteau-Ledouble S, El-Matbouli M (2020). The nature and consequences of co-infections in tilapia:A review. J. Fish Dis.

[27] Pelczar M.J, Chan E.C.S, Krieg N.R (2008). Microbiology.

[28] Li C, Tian Q (2011). Choice of chemicals to prevent or control saprolegniasis in songpu mirror carp eggs. Chin. J. Fish.

[29] Das S.K, Murmu K, Das A, Shakuntala I, Das R.K, Ngachan S.V, Majhi S.K (2012). Studies on the identification and control of pathogen *Saprolegnia* in selected Indian major carp fingerlings at mid-hill altitude. J. Environ. Biol.

[30] Neish G.A (1977). Observations on saprolegniasis of adult sockeye salmon, *Oncorhynchus nerka* (Walbaum). J. Fish Biol.

[31] Willoughby L.G, Roberts R.J (1992). Towards strategic use of fungicides against *Saprolegnia parasitica* in salmonid fish hatcheries. J. Fish Dis.

[32] Mazurkiewicz-Zapałowicz K, Twarużek M, Grzonka J, Kurzydłowski K.J (2015). The effect of magnetic field on *in vitro* development of fungus-like organisms *Saprolegnia parasitica* on selected microbiological media. Electron. J. Pol. Agric. Univ.

[33] Molina F.I, Jong S.C, Ma G (1995). Molecular characterization and identification of *Saprolegnia* by restriction analysis of genes coding for ribosomal RNA. Antonie Leeuwenhoek.

[34] de la Bastide P.Y, Leung W.L, Hintz W.E (2015). Species composition of the genus *Saprolegnia* in finfish aquaculture environments, as determined by nucleotide sequence analysis of the nuclear rDNA ITS regions. Fungal Biol.

[35] Sandoval-Sierra J.V, Martín M.P, Diéguez-Uribeondo J (2014). Species identification in the genus *Saprolegnia* (Oomycetes):Defining DNA-based molecular operational taxonomic units. Fungal Biol.

[36] Chauhan R, Bhat M.H (2014). Histopathological manifestations in commercially important fish, *Clarias batrachus* (L.) found infected with *Saprolegnia diclina*. Indo Am. J. Pharm. Res.

[37] Songe M.M, Willems A, Wiik-Nielsen J, Thoen E, Evensen O, van West P, Skaar I (2016). *Saprolegnia diclina* III A and *S. parasitica* employ different infection strategies when colonizing eggs of Atlantic salmon, *Salmo salar* L. J. Fish Dis.

[38] Refai M.K, Mohamed A, Amany M (2010). The assessment of myotic settlement of freshwater fishes in Egypt. J. Am. Sci.

[39] Copland J.W, Willoughby L.G (1982). The pathology of *Saprolegnia* infections of *Anguilla anguilla* L. elvers. J. Fish Dis.

[40] Hussein M.M.A, Hatai K (2002). Pathogenicity of *Saprolegnia* species associated with outbreaks of salmonid saprolegniosis in Japan. Fish. Sci.

[41] Bootsma R (1973). Infections with *Saprolegnia* in pike culture (*Esox lucius* L.). Aquaculture.

[42] Shin S, Kulatunga D.C.M, Dananjaya S.H.S, Nikapitiya C, Lee J, De Zoysa M (2017). *Saprolegnia parasitica* isolated from rainbow trout in Korea:Characterization, anti-saprolegnia activity and host-pathogen interaction in zebrafish disease model. Mycobiology.

